# Preparation and Publication of the Medical and Surgical History of the War

**Published:** 1868-06

**Authors:** 


### Preparation and Publication of the Medical and Surgical History of the War.

It will be seen by the following extract from Congressional proceedings, that $30,000 have been appropriated for the preparation and publication of the Medical and Surgical History of the War. The work is to be compiled and completed by Dr. J. H. Baxter, which is sufficient guarantee that it will be made in every respect as perfect as possible:

§ 3. *And be it further enacted*, That of the appropriation of $60,000 for publishing the medical and surgical history of the rebellion and the medical statistics of the Provost Marshal General's office, made in an act approved July 28th, 1866, $30,000 shall be devoted to the preparation and publication of five thousand copies of the medical statistics of the Provost Marshal General's Bureau, and that the work shall be compiled and completed by Assistant Medical Purveyor J. H. Baxter, under the immediate direction of the Secretary of War, and without the interference of any other officer.

Mr. Anthony. There was a proposition made to change that appropriation and to make it applicable to the preparation of the whole work. I believe, as the resolution was passed by Congress originally, this money was made applicable to the publication of the work; at any rate it was so construed by the Congressional Printer; and I have been trying for months to get a resolution passed through Congress to make the appropriation applicable to the preparation of the work, so that the ordinary appropriation for printing may be applicable to the publication of it. If those who have charge of this matter—I have not—have taken it into consideration, I do not wish to interpose any objection.

Mr. Conkling. If the Senator from Rhode Island will allow me, I desire to suggest to him that this proposition has no effect one way or the other upon the idea which he now submits. This does not touch at all the question whether money shall be appropriated to the preparation and not to the printing of the work, or whether it shall be applicable alike to the preparation and the printing. That question is left entirely untouched and open for the resolution of the Senator or any other treatment which the Senate may see fit to bestow upon it. This is simply a proposition that the appropriation shall be divided. It is directory in its nature and divides the appropriation precisely as I have the evidence in my hand to show both Houses intended that it should be divided originally as between the two works, namely, the statistics of the Provost Marshal General's Bureau, and the medical and surgical history of the war being separate things, and the purpose being to devote half of the appropriation to one and the other half to the other; but the language of the bill left it, as it was supposed, obscure, and this is simply to draw the dividing line.

The amendment was agreed to.

The bill was reported to the Senate as amended, and the amendments were concurred in.

The amendments were ordered to be engrossed, and the bili to be read a third time. The bill was read the third time, and passed.

